# Factors affecting delay in seeking treatment among malaria patients along Thailand-Myanmar border in Tak Province, Thailand

**DOI:** 10.1186/1475-2875-14-3

**Published:** 2015-01-07

**Authors:** Krit Sonkong, Sunisa Chaiklieng, Penny Neave, Pornnapa Suggaravetsiri

**Affiliations:** Faculty of Public Health, Khon Kaen University, Khon Kaen, Thailand; Department of Environmental Health Science, Faculty of Public Health, Khon Kaen University, Khon Kaen, Thailand; Department of Public Health, Faculty of Health and Environmental Sciences, Auckland University of Technology, Auckland, New Zealand; Department of Epidemiology, Faculty of Public Health, Khon Kaen University, Khon Kaen, Thailand; Tak Provincial Health Office, Tak, Thailand

**Keywords:** Delay in seeking treatment, Malaria, Risk factors, Hill tribe, Self-treatment, Social support

## Abstract

**Background:**

Malaria is a major health problem in Thailand, especially in areas adjacent to the borders of Myanmar. Delay in seeking treatment is an important factor in the development of severe complications, death and the transmission of the disease. This study aimed to investigate factors affecting delays in seeking treatment of malaria patients.

**Methods:**

A cross-sectional analytic study was conducted in 456 malaria patients along the Thailand-Myanmar border. Patients were selected by stratified sampling from 11 malaria clinics and five public hospitals in Tak Province, Thailand. Data were collected by the use of a structured interview questionnaire and from patient’s medical records.

**Results:**

The majority of patients were categorized with an ethnicity of ‘hill tribe’ (65.8%), followed by Thai (34.2%). Seventy-nine per cent of patients delayed seeking treatment. A simple logistic regression identified significant factors affecting delays in seeking treatment: people of “hill tribe” ethnicity; plasmodium species; self-treatment; visiting sub-district health promotion hospital/malaria post before visiting a malaria clinic or public hospital; and low to medium social support. After being subjected to multivariate analysis, factors significantly associated with the delay were “hill tribe” ethnicity (OR_adj_ = 2.32, 95% CI: 1.34-4.04); infection with *P.vivax* (OR_adj_=2.02, 95% CI: 1.19-3.41; self-treatment (OR_adj_ = 1.73, 95% CI: 1.04-2.85); and receiving a low degree of social support (OR_adj_ = 2.58, 95% CI: 1.24-5.35).

**Conclusions:**

Emphasis should be placed on need for early diagnosis and treatment in malaria patients as well as on ensuring the first facility for detection and treatment of malaria is a malaria clinic or public hospital, and the promotion of social support. These are especially important issues for the health of hill tribe people.

## Background

Malaria is a serious disease and a major public health problem, which causes the death of children and adults worldwide. Infection in humans is caused by a parasite belonging to the *Plasmodium* genus, and the species *Plasmodium falciparum* causes the majority of morbidity and mortality association with this disease [1–3].

In 2012, there were 207 million cases of malaria worldwide, with the majority (80%) occurring in the African region. Thirteen per cent occurred in Southeast Asia, and this represents a reduction in the prevalence of only 236 500 (14.4%) since 2000, despite targeted interventions to eliminate malaria in the region [1].

Despite being a country which has targeted malaria elimination as a public health priority, malaria remains a major health problem in Thailand, especially in areas adjacent to the borders of Myanmar and Cambodia [4–8]. Tak Province, comprising five districts along the Thailand-Myanmar border (Mae Sot; Phop Phra; Umphang; Mae Ramat and Tha Song Yang) has a particularly high prevalence of malaria. For example, between 2008–2010, the malaria morbidity rate per 100 000 population in this province was higher than in any other part of Thailand (1,400.78 in 2008, 1,606.06 in 2009 and 1,617.59 in 2010 per 100,000 population) [4, 6, 7], with and 50.4% of patients of Thai nationality and 29.2% migrants from Myanmar [8]. These high morbidity rates in border areas have been attributed to the geography (forest, hill and stream) which provide optimal conditions for the breeding of *Anopheles*, alongside high levels of migration across the borders of Thailand and Myanmar [4–8]. People of Thai nationality but who identify ethnically as ‘hill tribe’, including Karen or Hmong, live in these high-risk areas for malaria and they differ culturally from other Thais.

The World Health Organization has emphasized that early diagnosis and prompt treatment for malaria should occur within 24 hours of the onset of symptoms to decrease the risk of severe complications and onward transmission [3]. Appropriate treatment-seeking behaviour and easy access to health services are important components vital to its success [9]. Specifically, it is recommended that patients should seek medical treatment following the onset of fever**,** a common symptom of malaria [10]. It has been recognized that self-treatment may lead to delays in seeking treatment [9, 11, 12]. Such delay may mean patients develop severe complications within three to seven days of onset of fever [13]. However, in the case of falciparum malaria, these may occur within a few hours [3, 14].

Previous studies have identified a high prevalence (79.4%) of delay amongst patients in seeking malaria treatment in the five districts along the Thailand-Myanmar border in Tak Province [15]. However, no previous studies have clarified the factors that may influence this delay. The purpose of this study was therefore to investigate the factors affecting the delay in seeking treatment among malaria patients in this area. It is hoped that the findings can be used to assist in the development of more effective strategies to combat malaria in this area.

## Methods

### Study area and population

**Figure 1:**
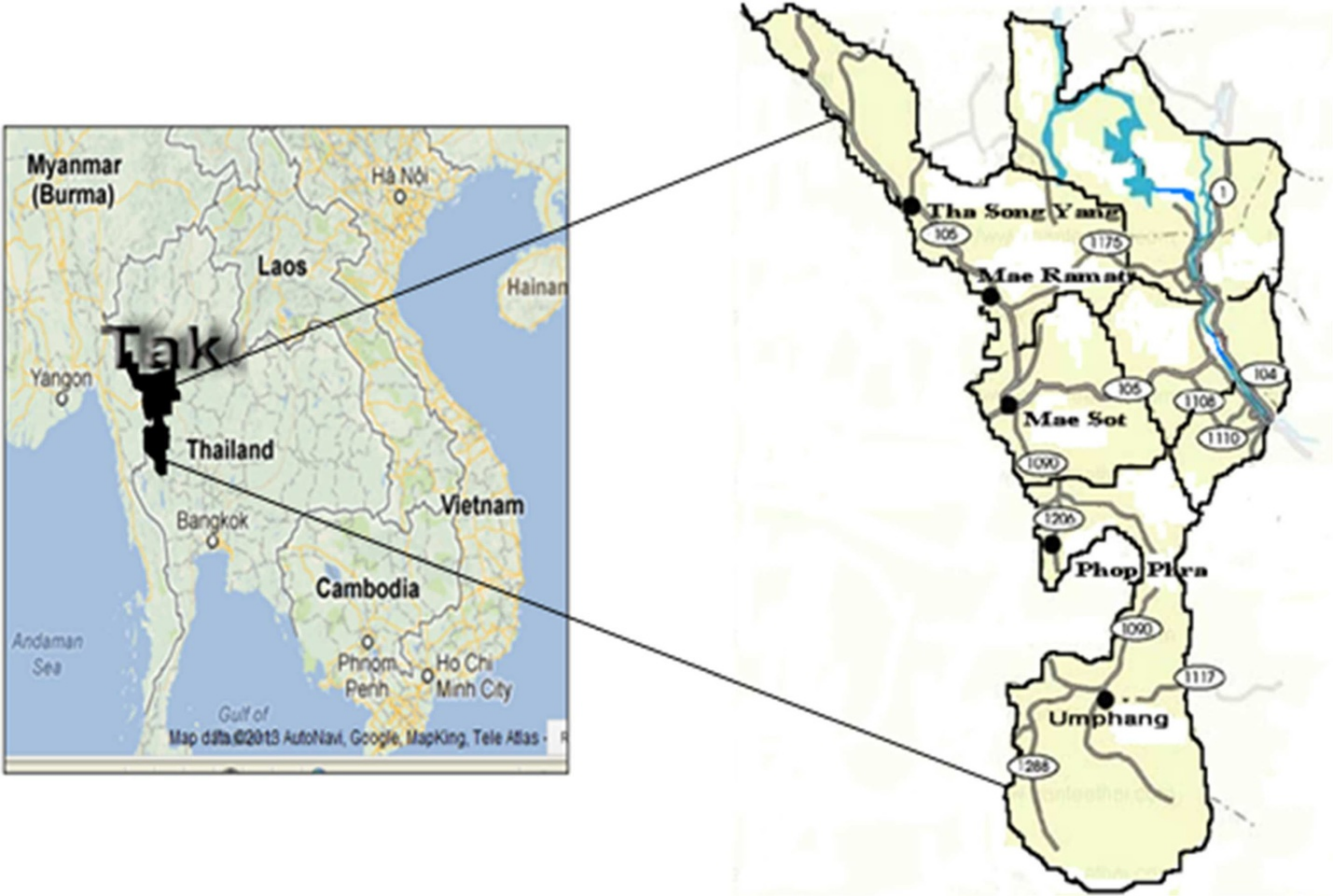
**The study site: five districts along Thailand-Myanmar border in Tak Province,** **Thailand.**

A cross-sectional analytic study was conducted along the Thailand-Myanmar border in Tak Province in five border districts with a high prevalence of malaria: Mae Sot, Phop Phra, Umphang, Mae Ramat, and Tha Song Yang (Figure 1).

### Selection and study participants

The participants were patients who had received laboratory-confirmed diagnoses of malaria as shown by the presence of any species of the *Plasmodium* parasite by thick blood film. They had been treated in 11 malaria clinics and five public hospitals between January 2011 and December 2011. The inclusion criteria were: patients of Thai nationality, over 15 years of age and resident in one of the five districts indicated in figure one. The exclusion criteria were: patients with a change of permanent residence after initial diagnosis as this might make follow-up patients difficult, and individuals with symptoms of mental illness which might affect their ability to provide accurate data. The participants were selected by stratified sampling from the clinics and hospitals, and the quota from each to be included was calculated as a proportion of the total number of patients treated in each hospital over the time period. These quotas ranged from nine to 125 patients for the 16 study sites. Recruitment at each clinic or hospital took place until the required number of respondents agreed to participate. All had been treated within one month of participation in the survey.

The sample size calculation of logistic regression analysis was applied to calculate the required number of participants [16], by using distance from home to health facility as the main independent variable of interest. The event rate of patients who delayed seeking treatment that had a distance of less than 3 km was 38.1% (P_1_ = 0.38), 61.9% (P_2_ = 0.62) was the event rate of patients who delayed seeking treatment that had distance of more than 3 km, and 31.0% (B = 0.31) was the proportion of patients who delayed seeking treatment that had distance of more than 3 km of the overall patients [17]. All of these proportions were taken into the formula for calculating sample size. By defining the power of the test at 90%, with 0.05% as a significance level, the minimum sample size was calculated as 456, after adjusting for variance and design effect.

### Data collection and analysis

Data were collected from patients by the use of a structured interview questionnaire and also from their medical records. Information received from these sources included sociodemographic characteristics, treatment-seeking behaviour, access of health services, and social support from family or community. Social support from family or community was defined as having receiving advice from one or more family or community members about how malaria is transmitted, its symptoms, complications, diagnosis, and treatment, and also having provided a respondent with either information, material, transport or money to attend a health clinic. The ten questions asked about social support were dichotomous, with a score of 1 for yes and 0 for no. Scores were categorized into three levels to attain a total score for social support (low <6; medium = 6-7; and high ≥8). Information relating to the date of the onset of symptoms and the date treatment at a malaria clinic or public hospital was sought was obtained from medical records and were used to classify whether patients had delayed seeking treatment. Delay in seeking treatment was defined as a period of more than 24 hours having passed between the onset of symptoms and treatment having been sought at a malaria clinic or public hospital. The interviews were conducted by people who were trained in each district to carry out the interview before the start of data collection.

All analyses were performed using the STATA 10.0 software package. Descriptive statistics were used to summarize the sociodemographic chararteristics. A bivariate analysis of the associations between the independent variables and delay in seeking treatment was performed using simple logistic regression. Checks were made for linearity, collinearity and interaction terms. Independent variables significantly related to delay in seeking treatment at the p-value ≤0.20 level in the bivariate analysis as well as gender and age were then subjected to a multiple logistic regression analysis to control for the effect of confounding variables. Statistical significance was defined as a p-value <0.05 while an adjusted odds ratio (OR_adj_) with 95% confidence interval (95% CI) was used as an indicator of the magnitude of association.

### Ethical approval and consent

This study was approved by the Committee of Human Research Ethics, Khon Kaen University, Thailand. Written consent was obtained from all patients before the interviews.

## Results

### Sociodemographic characteristics

The median age of the 456 participants was 34 years (range 15-80 years), and most were male (67.5%). In terms of educational background, 30.9% reported having received no formal education, 38.4% had attended primary school, and 30.7% had progressed beyond primary school. Fifty per cent of the subjects were employed in agriculture, 23.0% were self-employed, and 12.3% were students. For most patients, monthly incomes were more than 3,000 baht (35.8%) or less than 1,000 baht (27.6%) (median=2,000; range=0-25,000) (average monthly income of country=5,268 baht) [18], and their ethnicity of “Hill tribe” was Karen (62.5%) or Hmong (3.3%), followed by Thai (34.2%). Just over half (55.9) of infections were with *P.vivax,* 41.9% with *P. falciparum* and 2.2% with mixed infections of *P.falciparum* and *P.vivax.*

### Treatment seeking behaviour

Sixty four percent of participants had treated themselves before seeking treatment by taking an antipyretic available at home, tepid sponge or purchasing drugs from shop, 20.0% had previously sought treatment at a malaria clinic, 11.0% at a public hospital, 3.3% at a sub-district health promotion hospital (where malaria diagnosis facilities are not available) and 1.1% at a malaria post. Those who attended the malaria post were initially found to be parasitiologically negative for malaria, despite showing symptoms of this disease. The majority of patients (79.4%) sought treatment at a malaria clinic or public hospital after a period of 24 hours from the onset of symptoms. The median time for this was three days (range: 1-26 days).

### Factors affecting delay in seeking treatment

Significant factors influencing delay in seeking treatment identified by the bivariate analysis were; being of hill tribe ethnicity, infection with *P. vivax*, self-treatment, visiting a sub-district health promotion hospital or malaria post for diagnosis and treatment before visiting a malaria clinic or public hospital, and receiving only medium or low levels of social support from family or community (Table 1).

**Table 1:** **Factors associated with delay in seeking treatment by bivariate analysis (n = 456)**

Factors	n	Delay (%)	OR (95% CI)	p-value
**Sociodemographic**				
Gender				
Male	308	243 (78.9)	1.00	
Female	148	119 (80.4)	1.10 (0.67-1.79)	0.708
Age (years)				
<35	233	184 (79.0)	1.00	
≥35	223	178 (79.8)	1.05 (0.67-1.66)	0.822
Education level				
Primary school and above	315	247 (78.4)	1.00	
No education	141	115 (81.6)	1.22 (0.74-2.01)	0.443
Occupation				
Non- agriculture	228	178 (78.1)	1.00	
Agriculture	228	184 (80.7)	1.17 (0.75-1.85)	0.488
Monthly income (baht)				
≤2,000	231	184 (79.7)	1.00	
>2,000	225	178 (79.1)	0.97 (0.61-1.52)	0.886
Status				
Single	120	91 (75.8)	1.00	
Married	311	252 (81.0)	1.36 (0.82-2.26)	0.231
Divorce/separated	25	19 (76.0)	1.01 (0.37-2.77)	0.986
Ethnicity				
Thai	156	112 (71.8)	1.00	
Hill tribe	300	250 (83.3)	1.96 (1.24-3.12)	0.004*
History of illness with malaria				
Yes	385	307 (79.7)	1.00	
No	71	55 (77.5)	0.87 (0.47-1.61)	0.663
Species of infection				
*P. falcipalum*	191	138 (72.3)	1.00	
*P. vivax*	255	216 (84.7)	2.13 (1.34-3.39)	0.001*
*P. falcipalum & P. vivax*	10	8 (80.0)	1.54 (0.32-7.47)	0.595
**Treatment-seeking behaviour**				
Self-treatment				
No	164	121 (73.8)	1.00	
Yes	292	241 (82.5)	1.68 (1.06-2.66)	0.027*
Visiting traditional healer				
No	449	356 (79.3)	1.00	
Yes	7	6 (85.7)	1.56 (0.19-13.18)	0.679
Visiting private clinic				
No	439	347 (79.0)	1.00	
Yes	17	15 (88.2)	1.99 (0.45-8.85)	0.367
Previous visit to malaria clinic/public hospitals				
No	434	344 (79.3)	1.00	
Yes	22	18 (81.8)	1.17 (0.39-3.57)	0.773
Visiting sub-district health promotion hospital/malaria post				
No	380	295 (77.6)	1.00	
Yes	76	67 (88.2)	2.15 (1.03-4.48)	0.042*
**Accessibility of health services**				
Distance (km)				
≤3	181	139 (76.8)	1.00	
>3	275	223 (81.1)	1.30 (0.82-2.05)	0.268
Transport				
Private transport	322	250 (76.1)	1.00	
Bicycle/walking	58	47 (81.0)	1.23 (0.61-2.25)	0.565
Hired transport	76	65 (85.5)	1.70 (0.85-3.40)	0.131
Travel time (minutes)				
≤30	384	300 (78.1)	1.00	
>30	72	62 (86.1)	1.74 (0.85-3.53)	0.128
Cost of visit (Baht)				
≤60	229	175 (76.4)	1.00	
>60	227	187 (82.4)	1.44 (0.91-2.28)	0.117
**Social support**				
High	243	178 (73.3)	1.00	
Medium	114	96 (84.2)	1.95 (1.09-3.47)	0.024*
Low	99	88 (88.9)	2.92 (1.47-5.81)	0.002*
Receiving mass media messages about malaria				
Yes	401	315 (78.6)	1.00	0.239
No	55	47 (85.5)	1.60 (0.73-3.52)	

*The statistically significant identification at p-value < 0.05.

In the multiple logistic regression analysis, the factors significantly associated with a delay in seeking treatment were being of hill tribe ethnicity (OR_adj_=2.32, 95% CI: 1.34-4.04), infection with *P. vivax* malaria (OR_adj_ = 2.02, 95% CI: 1.19-3.41), self-treatment (OR_adj_ = 1.73, 95% CI:1.04-2.85), and receiving a low degree of social support (OR_adj_=2.58, 95% CI: 1.24-5.35) (Table 2).

**Table 2:** **Factors associated with delay in seeking treatment by multiple logistic regression (n = 456)**

Factors	n	Delay (%)	OR	OR _adj_ (95% CI)	p-value
Gender					
Male	308	243 (78.9)	1.00	1.00	
Female	148	119 (80.4)	1.10	0.88 (0.52-1.51)	0.653
Age (years)					
<35	233	184 (79.0)	1.00	1.00	
≥35	223	178 (79.8)	1.05	1.10 (0.65-1.89)	0.717
Ethnicity					
Thai	156	112 (71.8)	1.00	1.00	
Hill tribe	300	250 (83.3)	1.96	2.32 (1.34-4.04)	0.003*
Species of infection					
*P. falcipalum*	191	138 (72.3)	1.00	1.00	
*P. vivax*	255	216 (84.7)	2.13	2.02 (1.19-3.41)	0.009*
*P. falcipalum & P. vivax*	10	8 (80.0)	1.54	0.82 (0.17-4.01)	0.802
Self-treatment					
No	164	121 (73.8)	1.00	1.00	
Yes	292	241 (82.5)	1.68	1.73 (1.04-2.85)	0.033*
Social support					
High	243	178 (73.3)	1.00	1.00	
Medium	114	96 (84.2)	1.95	1.96 (1.00-3.20)	0.051
Low	99	88 (88.9)	2.92	2.58 (1.24-5.35)	0.011*

*Statistical significance was set at a p-value < 0.05.

## Discussion

Delay in seeking treatment is an important risk factor for severe complications and transmission of malaria, and remains a serious problem for patients living along the Thailand-Myanmar border in Tak Province. It was estimated that 79.4% of participants experienced a delay in seeking treatment. This is similar to previous estimates of the proportion of patients who delay seeking treatment, which have found that between 68.0 and 91.4% do so [19–23].

Ethnicity was a significant factor associated with a high rate of patient delay in seeking treatment. The delay was significantly higher in people of hill tribe ethnicity compared to other ethnic categories. The majority of hill tribe people in this study were Karen (62.5%). Previous studies have suggested that their diverse languages and beliefs in supernatural causes during an illness, resulting from current or past misdeeds of traditional healing, can be barriers to understanding modern western ideas and practices that might be related to a delay [24, 25]. Interestingly, the research presented here suggests that had only 1.5% of respondents had visited a traditional healer, whilst the majority had initially attempted self-treatment. The majority of participants reported having suffered previous malaria illnesses, and this may have made them feel confident in initially attempting to manage the illness themselves.

Infection with *P. vivax* was one factor associated with delay in seeking treatment. Infection with *P. vivax* malaria may cause less severe symptoms than *P. falciparum* and mixed infection [3]**,**[26]. However, it is worth highlighting that large proportions of those infected with both *P. falciparum* and *P. vivax* delayed seeking treatment (Table 1).

Self-treatment by taking ‘left-over’ drugs at home or drugs from a convenient shop is a first choice to relieve fever, as reported in many previous studies [10–12, 27, 28]. Patients may wait until symptoms become more serious before visiting a health facility. Some studies have indicated that self-treatment may occur particularly where initial symptoms experienced are mild, or during the initial period of illness [11, 29, 30]. However, it can quickly progress to the development of severe symptoms.

The decision of malaria patients to seek treatment at a health facility was not dependent on the distance from home, this is consistent with a study in northern Sri Lanka [11], but in contrast to those carried out in Ethiopia and Myanmar [17, 21]. The explanation might be that patients in this study area had easier access to convenient forms of travel than in Ethiopia and Myanmar [17, 21], either by motorcycles or cars. Nevertheless, travel by these means in hilly and remote areas can be difficult during the rainy season.

The findings showed that receiving low social support from family or the community was associated with a delay in seeking treatment. Malaria patients who received medium or high levels of social support may seek treatment rapidly following the onset of symptoms, and it may be a factor that helps malaria patients to understand the possible complications and severity of malaria occurring from self-treatment. It may serve to increase the perception of malaria as not being a routine illness, but one with a high risk of developing severe symptoms. The results reported from this study suggest that it is the personal intervention of friends and family, rather than mass media messages about malaria which are important. The costs of visiting a health facility, and educational level were not found to be statistically significant barriers to seeking treatment. However it should be noted that the median monthly income was less than the national median. One component of social support was receiving money from family or community members to access malaria treatment. Further exploration is needed of whether or how poverty impacts on decision-making about malaria treatment amongst ethnic groups living in border areas.

One limitation of this study was the classification between delay in seeking treatment and non-delay. This classification used medical records, which provided only the date of the onset of symptoms and date of seeking treatment at a malaria clinic or public hospital, and did not include the time of day this was sought. It is possible, therefore, that some patients who had an onset of symptoms early in one day, but who did not seek treatment until late the following day were misclassified as not having delayed treatment seeking. In addition, the study did not include those who moved residence frequently, these may have included migrants, and these may have different patterns of access to treatment for malaria. It is possible that some people infected with malaria were successful in treating themselves, or sought adequate treatment at a malaria post or health promotion hospital and these were not included in the study. However, it is likely that the study captured the most severe cases of malaria, as they attended a hospital, and it provides useful information about the factors which impact on delay in treatment seeking for these individuals.

## Conclusion

The multiple logistic regression analysis strongly confirmed that being of the ethnicity hill tribe and infection with *P. vivax* were significant factor associated with delay in treatment-seeking among malaria patients living in Tak Province. Initial self-treatment and low social support were other significant factors. The continuing problem of multidrug resistance, which may spread to other parts of Southeast Asia, means that malaria control in the Thailand-Myanmar border areas is likely to continue to be a public health priority. Emphasis should be placed on informing local communities about the need for accurate diagnosis and early treatment seeking in malaria patients and the promotion of social support based on assistance and advice as well as accurate knowledge about malaria.
